# Education as a tool for improving canine welfare: Evaluating the effect of an education workshop on attitudes to responsible dog ownership and canine welfare in a sample of Key Stage 2 children in the United Kingdom

**DOI:** 10.1371/journal.pone.0230832

**Published:** 2020-04-20

**Authors:** Anna Baatz, Katharine L. Anderson, Rachel Casey, Maria Kyle, Kirsten M. McMillan, Melissa Upjohn, Hollie Sevenoaks

**Affiliations:** 1 Education Department, Dogs Trust, London, United Kingdom; 2 Canine Behaviour and Research Department, Dogs Trust, London, United Kingdom; University of Lincoln, UNITED KINGDOM

## Abstract

One of the core objectives of many animal-welfare organisations is to achieve improvements in animal welfare through school education programmes. However, whilst many charities and organisations develop and deliver these educational activities, impact relating to specific animal welfare attitudes and behaviours remains largely undescribed. This study evaluated the effects of an hour-long dog welfare workshop delivered to children aged 7–11, evaluating 2732 learners in state primary schools across the UK. Two types of workshop were evaluated; “Be Dog Smart” (BDS) and “Responsible Dog Ownership” (RDO). This study assessed short-term impact on attitude outcomes, as a first step in developing a full education monitoring and evaluation framework. Learners within each class were randomly assigned to two groups; one completing an attitude-based questionnaire before (control) and the other after the workshop (treatment). Dog ownership status, age, gender, and social deprivation (measured as access to free school meals) were collected for all participants. Questionnaire scores were compared between treatment and control groups. Mean scores were significantly different (BDS p<0.001; Cohen’s D 0.65; RDO p<0.001; Cohen’s D 0.51) between control (BDS 13.57 ± 3.15; RDO 22.97 ± 4.78) and treatment groups (BDS 15.61 ± 3.10; RDO 25.47 ± 5.06) for both workshops, suggesting workshops effectively convey key messages and improve learner attitudes concerning dogs. Gender, age and social deprivation were found to significantly influence questionnaire responses. These findings contribute to a broader effort to improve canine welfare via childhood education while also demonstrating the feasibility of effective monitoring and evaluation during operational delivery of a schools workshop programme. Ongoing impact assessment is important in ensuring successful development, delivery and refinement of educational programmes to maximise the probabilty of positive changes in participants. Further work is needed to evaluate longer term impact, and ensure that desired influences on human behaviour change, and animal welfare, are achieved.

## Introduction

Many charitable (non-profit) organisations and individuals are working towards improving animal welfare. Despite the introduction of the United Kingdom’s (UK) Animal Welfare Act [1], animal cruelty and neglect remain a large and concerning problem [2].

Workshop-based education programmes for school age children are suggested to be a vital part of the solution to improving canine welfare, enabling learners to gain better understanding of dogs and behaving more appropriately around them [3]. The potential of influencing knowledge and behaviours around animal welfare issues in the next generation means that animal welfare charities commit considerable investment into these programmes, although animal welfare-related education is still often overlooked by governments.

The paucity of systematic scientific evaluation of programmes [4] means there is limited evidence as to how animal welfare education programmes impact children’s knowledge and attitudes. However, a recent study evaluated the impact of the SSPCA’s ‘Prevention through Education’ programme which highlighted a tendency for improved animal welfare conducive attitudes, knowledge, attachment and belief in animal minds following an animal welfare education intervention [5]. Other studies have demonstrated short term improvement in knowledge and / or attitudes following classroom-based education interventions in the United States, Italy and India [6–8]. Education programmes are particularly salient around dog bite prevention, given the health and welfare risks for both children and dogs associated with human directed aggression. A systematic review of 12 education interventions targeting dog bite prevention, using both classroom and home based techniques (e.g. video based vs. live-dog), concluded that education interventions have moderate efficacy in reducing the risk of dog bites to children, and that classroom-based lessons evidenced the most success in knowledge and behaviour changes [9]. One study evaluated the impact of an educational intervention through observation of pupils’ subsequent behaviour around a real dog; this suggested that an immediate change of behaviour is possible as a result of an educational intervention [10]. Recent discussions in the UK around dog control legislation have highlighted the importance of education in the reduction of dog bites and injuries. However these discussions have noted that there is currently insufficient evaluative evidence of the impact of such programmes, and more studies in this area are required [11,12].

As the long term impact of interventions is largely dependent on human behavioural change, assessment of impact can usefully employ the theories developed to aid understanding of human behaviour and decision making [13]. With the increasing interest in influencing human behaviour for a wide range of purposes, a framework to summarise characterising and designing behaviour change interventions has been described, in the formation of a hub termed the ‘behaviour change wheel’ [14]. This concept incorporates education as one of the interventions alongside the specific behaviours to be targeted. Taking a ‘Theory of Change’ (ToC) approach to assessing impact involves mapping clear causal pathways to identify all the underlying issues related to the prevailing problem and the changes and conditions required (the outcomes) to reach the end-goal. These outcomes may include intermediate and long-term stages of the component issues which are assumed to underly the end-goal. For education specifically, a clearly stated intended impact is needed, based on how education contributes to the organisational end-goal. A causal pathway between intermediate outcomes (e.g. knowledge acquisition) and long-term outcomes (e.g. behaviour change) helps to identify the necessary outputs (in this case, the type and content of the educational intervention) needed to reach these outcomes [15]. Thereafter a methodology for monitoring and evaluation (M and E) of these impacts is required. In this study, the Theory of Change (ToC) model was utilised to develop and evaluate the impact of workshops delivered by the Dogs Trust Education Officers across the UK.

**Fig 1:**
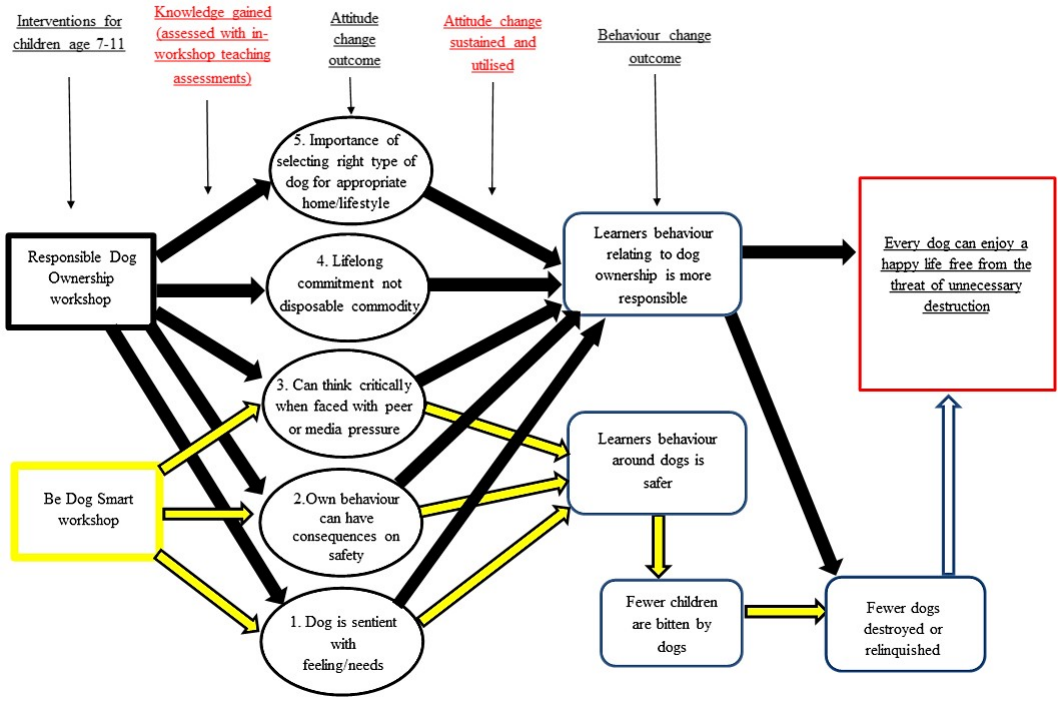
Dogs Trust education Theory of Change model. Theory of Change model for Dogs Trust educational workshop, aiming to develop attitude and behavioural outcomes that contribute to the Dogs Trust mission.

Dogs Trust is the largest dog welfare charity in the UK, and has delivered single event, in-school education workshops by a team of Education Officers (all certified teachers) across the UK since 2003, reaching thousands of learners a year [16]. Applying structured M and E processes to examine the programmes’ efficacy enables the organisation to understand the contribution of such activities towards achieving organisational objectives. Dogs Trust education workshops operate under a ToC model (Fig 1) where a large-scale end goal (Dogs Trust’s mission statement) is broken down into intermediate outcomes that connect in a causal chain. Assumptions underlying this causal chain need to be tested to validate the model.

Each Dogs Trust educational workshop is designed to work towards and evidence a learner demonstrating positive short-term change in core attitude outcomes. For Be Dog Smart (BDS) workshops the first three listed below are desired outcomes, for Responsible Dog Ownership, (RDO) workshops all five are targetted:

1. appreciation that dogs are sentient beings with feelings and needs,
2. understanding that their own behaviour can have an impact both on their own safety and overall dog welfare,
3. the ability to think critically and select information based on welfare, rejecting conflicting information from peers and media,
4. understanding that a dog is a lifetime commitment, not a disposable commodity,
5. understanding of the importance of selecting the right type of dog to suit their home and lifestyle.

Changes in these outcomes are assumed to create conditions necessary to cultivate subsequent long-term behaviour change conducive with both safer behaviours when around dogs with the associated potential to prevent dog bites and subsequent dog destructions, and behaviour supportive of more responsible dog ownership.

The BDS and RDO workshops evaluated in this study are designed for children aged 7–11 only, as this age range is suggested to be when specific cognitive abilities crucial to the outcome of interest develop [17,18]. The workshops have been designed in line with national curricular expectations for this age range particularly relating to the concept of responsibility, risk and use of critical thought [19,20].

The primary aim of this study was to assess the effect of Dogs Trust primary school education workshops on learner attitudes to dog ownership and dog interaction. As a secondary aim, the effect of other factors previously reported to influence these outcomes (gender, dog ownership, age and socioeconomic factors) were investigated. Our hypothesis was that children aged 7–11 would have a significantly enhanced attitude towards safety around dogs and responsible dog ownership after attending a Dogs Trust educational workshop as compared to reported attitudes prior to the event.

## Methods

### Ethical approval

Ethical approval for this study was granted by Dogs Trust Ethical Review Board in March 2018, reference number ERB004. Consent for data collection during workshops was initially gained from the Head of the School at time of booking. If deemed necessary by the school, individual parental consent was thereafter sought. A clear opt out option at each point was offered for those not wishing to be included. A second opt-out stage was delivered verbally by the Education Officer delivering the workshop first to the teacher of the class prior to the workshop commencing, and finally direct to the learners in the class.

### Questionnaire development

As no validated pre-existing tool was available for the specific attitude outcomes of our intervention (Fig 1), a new questionnaire was developed for this study (see S1 and S2 Files). This was based on published non-cognitive educational skills tools used to evaluate scores for similar interventions [21,22]. Pre-testing involved piloting the tool with 120 children to assess age suitability and ease of completion. The questions assessed attitudes that support the five short-term outcomes discussed above addressed within the workshops using a likert scale. Two questions were developed per outcome—hence the BDS questionnaire comprised 6 questions demonstrating:

1. Learners appreciated dog is sentient with feelings
2. Learners show understanding that their own behaviour can have an impact both on their own safety and overall dog welfare
3. Learners demonstrate the ability to think critically and select information based on welfare, rejecting conflicting information from peers and media

The RDO questionnaire comprised 10 questions addressing outcomes 1–3 as above plus questions 7–10 addressing outcomes 4 and 5 demonstrating:

4. Learners understand that a dog is a lifetime commitment, not a disposable commodity,
5. Learners understand the importance of selecting the right type of dog to suit their home and lifestyle.

Each question was scored from 0 (strongly disagree) to 4 (strongly agree), and therefore the maximum score in BDS workshops is 24 and in RDO 40, where a higher score reflects more positive attitudes. Four questions in the BDS, and seven questions in the RDO tool were randomly reversed to avoid guessing of ‘correct’ answers, and these scores are inverted accordingly, so that the higher scores remain as the more positive attitude. The following data were also collected, suggested by previous research to be potential confounding factors: the participant’s socio-economic status [23] measured by proxy as Free School Meal (FSM) (the provision of free meals at school for disadvanteged children under the Education Act 1996, in the UK) at class level %, current dog ownership status (measured at participant level) [24], age (measured by proxy as school year group at class level) and gender (measured at participant level) [25–27].

### Selection of study participants

During May-July 2018 twenty Education Officers working UK wide, each sampled eight classes. Four of these were allocated a BDS workshop and four a RDO workshop. Based on 80% power to detect an estimated score difference of 20% and allowing for class-level clustering at 4 per Education Officer per intervention type, the required sample was 7 children per group (i.e. 7 ‘treatment’ and 7 ‘controls’).

Education Officers normally book a selection of class workshops in each school visit they conduct, for operational reasons. For this analysis, for schools where more than one class was delivered, one class per school was randomly selected. Where logistically feasible, each Education Officer selected two of each Key Stage 2 year group (age 7–11 according to the English education system). To balance for gender as far as practicable, all schools included in the sample were co-educational.

### Data collection

Prior to collecting data in workshops all Education Officers were trained by the lead researcher (AB) in data collection methods, including minimising bias. Learners within each class were randomly assigned as number one or number two. After being assigned their number, learners were seated on opposite sides of the classroom to separate number 1s as the control (C) (pre-intervention) and number 2s as the treatment (T) (post-intervention) group. The between-subject methodology was favoured over a within-subject study design, to minimise social desirability biases and ’Hawthorne Effect’ [28]. Given the short duration of the intervention which was on average 60 minutes in length a within-subject approach would have resulted in participants responding twice to the same set questions only 60 minutes apart. Exposure to these questions immediately prior to the intervention could influence their focus on information transfer within the intervention. Randomisation into a control and treatment group ensured participants were exposed to the questionnaire only once, thus responses in the treatment group were not influenced by prior exposure.

Following randomisation, the Education Officer provided each learner in the control group with a copy of the questionnaire to complete in silence in their own time. Once questionnaires were collected, the workshop was delivered to the whole class with the two groups remaining in their allotted classroom sides (see S3 and S4 Files for intervention material). On completing the workshop, the treatment group were given questionnaires and allowed to complete these in silence before collection. Care was taken to minimise social desirability response bias or the temptation to copy one another by the Education Officer verbally clarifying to the class that the questionnaire was anonymous and there was no right or wrong answer. Teachers and Teaching Assistants were asked not to aid pupils in the completion of questionnaires unless to address literacy or English as an additional language difficulty. In the event that the Education Officer suspected the learner was being guided in their answers the questionnaire was marked and subsequently destroyed without the learner being made aware, in order to avoid embarrassment. The completed questionnaires for the treatment and control groups were collected into separate envelopes, clearly marked and sent directly to Dogs Trust Head Office for data entry.

### Data analysis

Statistical analysis was conducted using IBM SPSS v25. Data were tested for normality graphically using Q-Q plots and histograms, and the appropriate parametric or non-parametric tests used. Each BDS and RDO workshop treatment group was analysed compared to their respective control group. An independent t-test or Mann-Whitney, where data assumptions were not met, was used to test whether there was a statistically significant difference (significance set at p<0.05) between the attitude scores achieved by the treatment versus the control group for each individual question and the overall score (between-subject). The mean scores and standard deviation were used to quantify the treatment effect size using Cohen’s D.

Using R (version 3.6.2; R Core Team 2019) [29] the relationship between free school meal (FSM) percentage, year group, dog ownership, gender and treatment intervention (variables defined in Table 1) and total attitude score were tested by creating two separate generalised linear mixed models (implementing a Gaussian error structure), for Be Dog Smart and Responsible Dog Ownership respectively. Model assumptions were tested graphically and collinearity was tested between variables using variance inflation factor (VIF). All logical and feasible hypotheses and subsequent models reflecting these were developed, creating a set of 17 candidate models constructed to test for relationship of the predictor variables and score outcome for both Be Dog Smart and Responsible Dog Ownership workshops (Supplementary files 5 and 6). Possible non-independence of learners was accounted for by including classroom as a random effect to control for the influence of classroom level factors on total score, and all other predictor variables (listed above) and logical interactions were included as fixed effects. Using Akaike information criterion (AIC) model selection was conducted to identify a top model set that best supported the data, using a delta 6 cut-off (all models within 6 points of the top model were deemed to have comparable support). A ‘nesting rule’ was applied whereby models that fell within 6 points of the best-fit model, and only those that were less complex than the top model, were retained in the top model set [30,31]. For Be Dog Smart, the top model set contained 4 models overall, however 3 were removed due to being more complex than the top model (S5 File) and for Responsible Dog Owneship, the top model set contained 2 models overall, however 1 was removed due to being more complex than the top model (S6 File). As only one model remained in both analyses, model-averaging was not suitable and consequently all reported results are based on the top model.

**Table 1:** Definitions and characteristics of the variables used within the statistical analysis.

Variable	Variable Type and Definition
Free School Meal Percentage (FSM%)	Free school meal percentage for participant’s class. Categorical (0–20%, 21–40%, 41–60%, 61%+)
Year Group	Year group of participant. Categorical (Year 3,4,5 and 6)
Gender	Gender of participant. Categorical (Male, Female and Rather Not Say)
Dog Ownership	Dog ownership status of participant. Categorical (Dog owner, Non dog owner)
Treatment	Random assignment of learner in to either treatment or control group. Categorical

## Results

A total of 2732 participant responses were used in the analysis, summarised below in Table 2. Summary descriptive statistics for both workshops are shown in Table 3. All questionnaires were included in the t-test/Mann-Whitney analyses. However, in analysis of effects of predictor variables (gender, FSM%, year group and dog ownership), cases with incomplete data were omitted (recorded in Table 3 as ‘Missing data’). The number of questionnaires omitted due to potential inaccuracies during data collection was not recorded. Tests for collinearity showed little correlation between variables with all VIF scores being below 1.5.

**Table 2:** Summary statsitsics for study participants.

	Be Dog Smart	Responsible Dog Ownership
Total Number of Children	1253	1479
*Controls*	*632*	*753*
*Treatment*	*621*	*726*
Number of Classes	49	59
Number of Schools	49	59

**Table 3:** Variable summary statistics for Be Dog Smart workshop and Responsible Dog Ownership workshops.

Variable Type	Be Dog Smart		Responsible Dog Ownership	
	Control	Treatment	Control	Treatment
**Free School Meal (%)**				
0–20	330	330	437	418
21–40	220	217	225	217
41–60	61	51	79	76
61+	21	23	13	15
**Year Group**				
3	189	156	191	186
4	120	133	188	170
5	193	175	165	160
6	158	157	210	210
**Dog Ownership**				
Dog owned	236	238	297	284
No dog owned	370	358	457	441
Missing data	26	25	-	1
**Gender**				
Male	290	284	355	293
Female	274	263	336	361
Rather not say*	10	20	20	30
Missing data	58	54	43	42

*NB for regression analysis rather not say was considered as missing data and omitted

### Be Dog Smart

#### How effective is a single Be Dog Smart workshop in improving children’s attitudes towards responsible dog ownership?

The treatment group had significantly higher scores than the control group (higher score represents more positive attitudes) overall (control 13.57 ± 3.15, treatment 15.61 ± 3.10; p<0.001) suggesting that BDS workshops have a significant positive effect on children’s attitudes towards dogs. The difference was also significant for all individual questions except question 4 (Table 4). The effect size was large (Cohen’s D 0.65), whereby the score of an average participant in the treatment group exceeds 73% of the scores of the control group. When picked at random there is a 66% chance of treatment participant scoring higher than a member of the control group.

**Table 4:** Comparison of mean results of individual question and total scores, between control (pre-intervention) and treatment (post-intervention) groups for Be Dog Smart workshops.

BDS Tool Question	Control group			Treatment Group			Mean Difference	Significance (p value)	Effect Size (Cohens D)
	N	Mean Score	Standard deviation	N	Mean Score	Standard Deviation			
1	632	3.39	0.85	621	3.56	0.80	0.17	<0.001	0.21
2	632	2.09	1.07	621	2.55	1.02	0.46	<0.001	0.44
3	632	1.64	1.04	621	2.29	1.15	0.65	<0.001	0.58
4	632	2.91	1.03	621	3.02	0.97	0.11	0.093	0.11
5	632	2.02	1.17	621	2.29	1.21	0.27	<0.001	0.23
6	632	1.52	1.15	621	1.91	1.18	0.39	<0.001	0.33
Total Score	632	13.57	3.15	621	15.61	3.098	2.04	<0.001	0.65

#### Factors predicting ‘Be Dog Smart’ attitude scores

The top model, reported below, included the variables treatment (intervention) and year group. Class-level factors were shown to explain 7% of the variance within total score. As described above, being part of the treatment group saw an increase in total score compared to the control group, and older year groups (years 5&6) were shown to score higher than younger year groups (Table 5).

**Table 5:** The top model multi-level regression analysis results for effect of treatment intervention and year group on total score (n = 1105).

**Random Effects**			
**Group**	**Variance**	**Standard Deviation**	
Class (n = 46)	0.65	0.81	
Residual	8.48	2.91	
**Fixed Effects**			
**Variable**	**Coefficient**	**Standard Error**	**t value**
Constant	13.10	0.311	42.09
Control	Reference	-	-
Treatment	1.96	0.18	11.15
Year 3	Reference	-	-
Year 4	0.06	0.45	0.154
Year 5	1.38	0.40	3.420
Year 6	0.70	0.41	1.71

### Responsible Dog Ownership

#### How effective is a single Responsible Dog Ownership workshop in improving children’s attitudes towards responsible dog ownership?

The treatment group had significantly higher scores than the control group (higher score represents more positive attitudes) overall (control 22.97 ± 4.78, treatment 25.47 ± 5.06; p<0.001), suggesting that RDO workshops have a significant positive effect on children’s attitudes of responsibility towards dogs. The difference was also significant for all individual questions except question 4 (Table 6). The Cohen’s D effect size for total score was 0.51, whereby the score of an average participant in the treatment group exceeds 69% of the scores of the control group. When picked at random there is a 64% chance that a participant from the treatment group will have a higher score than someone picked randomly from the control group.

**Table 6:** Comparison of mean results of individual question and total scores, between control (pre-intervention) and treatment (post-intervention) groups for Responsible Dog Ownership workshops.

RDO Tool Question	Control group			Treatment Group			Mean Difference	Significance (p value)	Effect Size
	N	Mean Score	Standard deviation	N	Mean Score	Standard Deviation			
1	754	3.37	0.84	725	3.44	0.83	0.07	0.038	0.08
2	754	2.19	1.05	725	2.35	1.02	0.16	0.004	0.15
3	754	1.80	1.13	725	2.03	1.08	0.23	<0.001	0.21
4	754	2.88	1.02	725	2.93	1.02	0.05	0.286	0.04
5	754	1.96	1.23	725	2.30	1.22	0.34	<0.001	0.54
6	754	1.65	1.11	725	2.06	1.17	0.41	<0.001	0.36
7	754	2.37	1.29	725	2.59	1.28	0.22	<0.001	0.17
8	754	1.95	1.24	725	2.45	1.11	0.50	<0.001	0.42
9	754	1.77	1.36	725	2.08	1.42	0.31	<0.001	0.22
10	754	3.03	0.97	725	3.22	0.94	0.19	<0.001	0.20
Total	754	22.97	4.782	725	25.47	5.061	2.50	<0.001	0.51

#### Factors predicting ‘Responsible Dog Ownership’ attitude scores

The top model, reported below, included the variables treatment (intervention), year group, FSM% and gender. Furthermore, the model contained an interaction between gender and year group. Class-level factors were shown to explain 9% of the variance within total score. Being part of the treatment group again saw an increase in total score compared to the control group. Females had higher attitude scores than males, except for males in Year 6 who scored higher than females, and older year groups (years 5&6) were shown to score higher than younger year groups, with the exception of males scoring lower in year 4 & 5 than year 3. Finally higher FSM percentage groups scored lower in comparison to 0–20% FSM (Table 7).

**Table 7:** The top-model multilevel regression analysis results for effect of free school meal %, year group and gender on total score (n = 1342).

**Random Effects**			
**Group**	**Variance**	**Standard Deviation**	
Class (n = 58)	1.88	1.37	
Residual	19.07	4.37	
**Fixed Effects**			
**Variable**	**Coefficient**	**Standard Error**	**t value**
Constant	21.95	0.54	40.92
Control	Constant	-	-
Treatment	2.49	0.24	10.38
Year 3	Constant	-	-
Year 4	1.42	0.70	2.03
Year 5	2.65	0.71	3.72
Year 6	3.45	0.68	5.10
Female	Constant	-	-
Male	-0.71	0.48	-1.47
FSM 0–20%	Constant	-	-
FSM 21–40%	-1.48	0.48	-3.08
FSM 41–60%	-0.39	0.73	-0.54
FSM 61%+	-0.90	1.67	-0.54
Gender*Year			
Male:Year 4	-0.65	0.70	-0.93
Male:Year 5	-0.67	0.71	-0.95
Male:Year 6	0.76	0.65	1.17

## Discussion

This study identified a significant difference between treatment and control scores for children attending Dogs Trust ‘Be Dog Smart’ and ‘Responsible Dog Ownership’ primary school workshops. We also identified an influence on questionnaire scores for gender (females scoring higher than males in RDO) and age (with older age groups scoring higher in both workshops). For social deprivation, results highlight that classes with a lower percentage of children receiving free school meals, often produce higher attitude scores within RDO workshops. Additionally, this study aimed to assess the feasibility of implementing a monitoring framework, using a controlled trial design to evaluate the impact of school workshops on children’s knowledge and attitudes about dogs within the constraints of normal operational requirements. This was intended to inform future Dogs Trust monitoring activities and to assist the development of assessment processes for interventional programmes delivered by other stakeholders involved in animal welfare education both in the UK and worldwide.

The aim of Dogs Trust’s Education workshops is to improve child learners’ attitudes towards dogs, and knowledge of responsible dog ownership. The results of this study are encouraging, indicating a significantly increased score (suggesting a more positive attitude) immediately following the delivery of the workshop to 7–11 year olds in the treatment group for both workshops as compared with the control group. There is an assumption in this work, which needs testing, that a change in attitude leads to behavioural change. Further studies are needed to investigate both long-term attitude, knowledge and behavioural changes whilst controlling for counfounding factors. Responses to question 4 (‘It is generally how I behave around a dog, that makes a situation safe or dangerous’) in the questionnaire were not significantly different between control and treatment scores for either workshop. During trialling phases, the tool was tested for feasibility only, and therefore ease of understanding or interpretation of question 4 may need reviewing. Whether question 4 is meaningiful to the participants and reflects the content delivered during the workshop may also need reviewing. Full scale development and validation of the tool [32] is required for future studies and to reassess inconsistencies. This will be incorporated into future trials. However, evidence suggests internal inconsistencies within tools are common in interventions involving children [33], and therefore question inconsistencies may be unavoidable.

The study also aimed to investigate possible variables that may influence the attitude of children after workshops. Previous research suggests dog ownership during childhood can result in greater empathy and prosocial attitudes in to adulthood [24] and that adulthood attitudes towards animal welfare positively correlate with pet ownership [34,35]. No influence on scores was detected between dog owners and non-dog owners in our study. This is similar to a previous study which found no difference between owners’ and non-owners’ empathy levels [36]. This suggests that education is equally important for all individuals, regardless of their pet ownership status.

Previous research has highlighted how socioeconomic influences can result in differences in knowledge attainment throughout education [23]. Prevalence of dog bite injuries resulting in hospitalisation have been suggested to be higher in lowest income areas measured in both urban and rural locations. We recorded the free school meal (FSM) percentage for each class, as a proxy indicator of socio-economic status [23]. For BDS, no relationship was detected. However, in RDO results, those with a lower FSM% scored higher than those with higher FSM%. The BDS and RDO workshops have similar participant profiles, therefore these mixed findings require further investigation to understand the root cause for the difference, including reviewing the structure of the two workshop types. A previous study proposed that education and prevention efforts should be suitably and carefully tailored and focused on particular areas/target groups [37] but the difference in the effect of socioeconomic status on outcome in our study suggests that the relationship between animal welfare education outcomes and socioeconomic status is not necessarily straightforward. Females demonstrated greater improvement in attitudes than males in our analysis for RDO workshops, which is not an uncommon finding [25], with previous studies finding females more likely to be more sympathetic towards animal welfare [26,27] and further develop recognition of internal emotions and sentience of animals [38]. Older children (years 5 and 6) scored significantly higher than younger children (years 3 and 4). However, the biggest effect was observed in the younger children. This combination of impact findings emphasises that ‘children’ as an audience should not be considered homogeneous.

An intrinsic consideration of likely influence on the outcomes should be the age-associated cognitive capabilities of the children receiving the intervention. Studies conducted with children aged under 7 suggest very limited evidence that a change in behaviour is likely to manifest in children this age as a result of gaining knowledge [39]. However more promisingly with interventions delivered to children aged over 7, there is evidence that learners can both sustain the change in attitudes at least a year after the intervention [40], and can demonstrate a change in actual behaviour around dogs [10]. Younger children may be more receptive of educational interventions in general, potentially due to the fact that they simply have more to learn [41]. With older children, there is also evidence that a standalone workshop delivered by an external organisation educator can have an even greater effect on childrens’ attitudes applying to behaviour change than a scheme of learning delivered by the children’s own teacher [42]. This is promising given the extent to which this ‘light touch’ single workshop approach is utilised by many animal welfare education programmes, amidst calls to integrate animal welfare into the UK curriculum [43].

Humans’ treatment of and feelings about animals are heterogeneous and subject to multiple personal and external influences. Animal welfare education programmes may be delivered in a multitude of countries and contexts, so a single programme’s causal framework or evaluations will only ever serve as marginally empirical. Effects and influences may differ from one context to another. Iterative M and E is vital to show whether what is being delivered is effective and to facilitate appropriate modifications of work programmes when objectives are not being met. Further to this, educational M and E should be viewed as a continual process of development to enhance education programs to optimise productivity with the aim of achieving human behaviour change in favour of animal welfare. Using structured monitoring of education activities in a large organisation facilitates identification of teams which are particularly successful, so that their approach can be shared across the organisation. Any unexpectedly lower contributions can be assessed to identify obstacles to achievement which can be addressed by changes in programme design and/or delivery. It also enables demonstration of effective use of supporters’ funds towards achievement of the ultimate goal.

Two key questions remain for an animal welfare education programme undertaking such a proof of concept evaluation:

1. Will the immediate effect of the treatment fade out before reaching the long term outcome?

While there is some evidence an improvement in animal welfare conducive attitudes can be sustained at least for a year [40], the heterogeneity of this field and associated differing outcomes warrants an ‘action research’ approach whereby each programme conducts their own longitudinal assessments allowing for maximum external validity of evaluative findings [44]. Should the change in attitudes associated with workshop attendance fade out quickly, a resulting change in behaviour is unlikely.

2. Will the change in attitudes that can be evidenced result in a change in behaviour?

A change in attitudes may be viewed as an intrinsic causal step towards the achievement of behaviour change, but it cannot be assumed that interventions that influence attitude necessarily lead to behavioural change [45]. For example, a sizeable treatment effect on responsible parenting attitudes was observed when an infant simulator doll was used as an educational intervention aimed at reducing teenage pregnancy [46]. However almost a decade later Brinkman et al [47] conducted a large scale RCT of the behaviour change arising from this intervention and found the treatment group were significantly more likely to become pregnant in their teenage years. The intervention was effective at changing attitudes but counter effective at changing behaviour—a salient reminder that making an assumption of links between short term changes and long term outcomes is risky. Hence, although this paper identifies a change in attitude subsequent to a single animal welfare education intervention, it cannot be assumed that behaviour change necessarily follows. Nevertheless, this paper demonstrates the feasibility of developing a protocol of M and E for animal welfare education programs, and is a starting point for further work investigating longer term influences on behaviour change. The heterogeneity of audience and contexts means that local M and E in each programme is essential to build understanding of factors affecting success in different contexts. Our own trial was relatively inexpensive and logistically feasible with minimal disruption to an already existing educational workshop process. It did not require substantial in depth training or additional skills for wider education team members. The methodology was relatively easy to follow and required little practical adaptation to what an existing education programme was undertaking in schools. Some level of expertise in impact assessment methodology and statistical approaches is necessary to develop the framework for this type of programme, and embedding these skills in the management or staffing of programmes to evaluate impact is an important consideration for animal welfare organisations.

### Limitations

This study only investigated short-term effects of an educational intervention on measures of attitudes and knowledge. Whilst the intervention had a positive outcome, this does not directly demonstrate the behaviour change this programme seeks to achieve. The possibility of ‘fade-out’ post-intervention, and how this is affected by the age at which children participate remain to be investigated. This study involved a convenience sample of schools throughout the UK, with purposive sampling of schools within the available population to facilitate inclusion of a range of values for variables of interest as far as possible, depending on invitations received from schools. Randomisation was only feasible at participant level within a given class. Since schools had to have requested the actual intervention before being invited to participate within this research study, an inherent selection bias arising from schools that wish to host a canine welfare educational intervention potentially not being representative of the general population of schools is unavoidable.

It should be noted that no valid pre-existing tool was relevant to the specific outcomes of our intervention and therefore our own tool was created. Pre-testing involved piloting the tool with 120 children to assess age suitability and ease of completion. Therefore, the inconsistencies in our results may be explained by the lack of validity and reliability of the tool. However, evidence suggests inconsistencies are common in interventions involving children [33], therefore this may not be avoidable. Full validation of the tool is required for future studies and the inconsistencies reassessed, this will be monitored across future trials. The data are also subject to potential biases, relating to social desirability bias and hawthorne effect, where participants in the study may have responded to the tool in a manner they consider to be more accepted as the correct answer [28,48]. Care was taken to minimise such biases by training the delivering team to clearly communicate to learners how important ‘honest’ answers were over ‘correct’ answers, and through using a between-subject versus a with subject study design. Care was taken to ensure that constraints involving the pupils’ ability to complete the questionnaires, such as literacy ability or English as an additional language, were identified without causing any embarrassment to the individual participant. Pupils were advised that if they found the questionnaire too hard to read and complete alone, that it was not a problem and to simply let the Education Officer or their teacher know by raising their hand. In the event of occurrence, the pupil’s questionnaire was discreetly taken from them and omitted. As this took place in the classroom the number of these omitted questionnaires was not recorded, they were discarded on the day and not included in this study. It should be noted that this could therefore result in selection bias favouring pupils with greater literacy or language skills, however due to practicalties of conducting research in schools this method of approach was deemed the most appropriate.

## Conclusion

Our study concludes that the Dogs Trust education programs effectively communicate our key messages as we found a significant difference between the scores of the control and treatment groups, whereby those that received the workshop recorded more positive attitudes towards dogs. We identified that year group (BDS and RDO), gender (RDO) and FSM% (RDO) significantly influenced total score, and therefore highlighted the need for appropriately tailored workshop content for the target audience. We also demonstrate that monitoring of existing programs is feasible, and forms a basis for longer term studies. The findings of impact assessments can increase the likelihood of delivering successful educational programmes that result in positive changes which improve animal welfare. A causal framework such as a ToC can be utilised as a first step for education programmes’ development of an M and E structure. There is a need for more structured M and E processes to be placed around animal welfare education, and it is important for each programme to develop monitoring relevant to their own context of activity. Reducing bias and randomising interventions as much as possible is an important element for evaluating the impact of these types of programmes, and ensuring collaboration with researchers familiar with these constraints is an important consideration. Longitudinal research of educational interventions and human behaviour change evaluation is also warranted to inform the animal welfare sector about the effectiveness of educational intervention in supporting behaviour change both in the short and longer-term.

## Supporting information

S1 File Tool used for data collection of attitudes towards safety around dogs. (DOCX)

S2 File Tool used for data collection of attitudes toward responsible ownership. (DOCX)

S3 File Lesson plan material for Be Dog Smart workshops for KS2 learners. (DOCX)

S4 File Lesson plan material for Responsible Dog Ownership workshops for KS2 learners. (DOCX)

S5 File Akaike’s information criterion candidate models for explaining effect of predictor variables on total attitude scores for a Be Dog Smart workshop, controlled at class level. *k*, number of parameters; *lokLik*, log likelihood; *AIC*, Akaike’s information criterion; *ΔAIC*, difference in AIC compared with the model with the lowest AIC; *w*_*i*_, model weight; *Retained*, models within delta-6 AIC are not retained if they are more complex versions of nested models with better AIC support; *Treatment*, Random assignment of learner in to either treatment or control group; *FSM*, Free school meal percentage for participant’s class; *Year*, year group of participant; *Gender*, gender of participant; *DO*, dog ownership status of participant.
(DOCX)

S6 File Akaike’s information criterion candidate models for explaining effect of predictor variables on total attitude scores for a Responsible Dog Ownership workshop, controlled at class level. *k*, number of parameters; *lokLik*, log likelihood; *AIC*, Akaike’s information criterion; *ΔAIC*, difference in AIC compared with the model with the lowest AIC; *w*_*i*_, model weight; *Retained*, models within delta-6 AIC are not retained if they are more complex versions of nested models with better AIC support; *Treatment*, Random assignment of learner in to either treatment or control group; *FSM*, Free school meal percentage for participant’s class; *Year*, year group of participant; *Gender*, gender of participant; *DO*, dog ownership status of participant.
(DOCX)
