# Supplementary material for: Education as a tool for improving canine welfare: Evaluating the effect of an education workshop on attitudes to responsible dog ownership and canine welfare in a sample of Key Stage 2 children in the United Kingdom
Source: PLoS One. 2020 Apr 20;15(4):e0230832. doi: 10.1371/journal.pone.0230832 (PMC7170237; doi:10.1371/journal.pone.0230832)

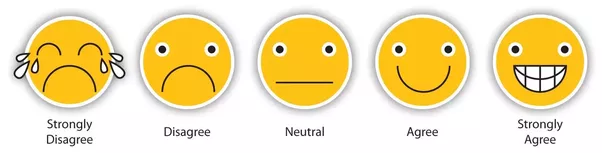
**Please only tick one answer for each question:**

I am a: (tick one) **Boy** **Girl** **Rather not say**

I have a dog at home: (tick one) **Yes** **No**

1. **Dogs are able to feel things such as pain, worry and fear**


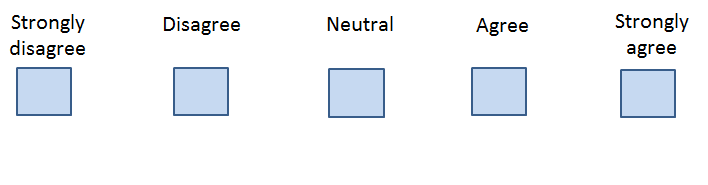


1. **Dogs will forgive us for everything and never react badly**


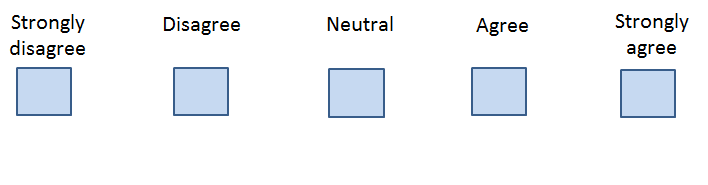


1. **Most dogs are friendly and would never bite**


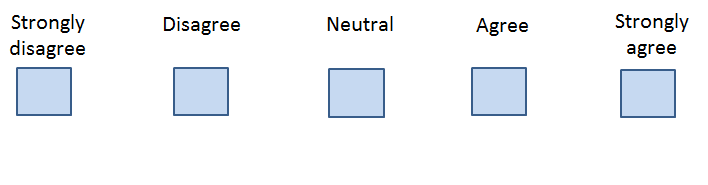


1. **It is generally how I behave around a dog that makes a situation safe or dangerous**


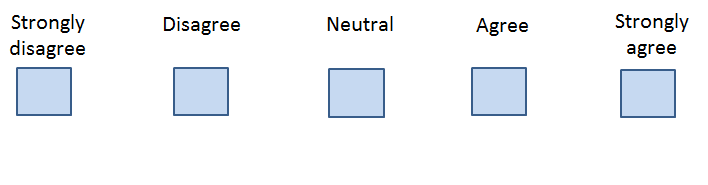


1. **When learning about dogs you should always follow advice you see on TV or the internet**


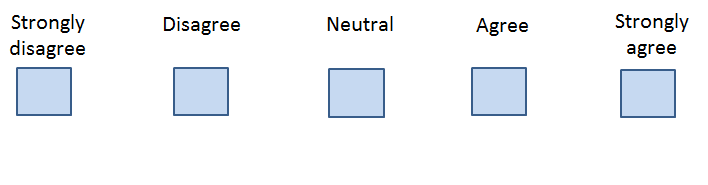


1. **I should always follow the advice of my family and friends, even if I believe there is a kinder choice that I could make**


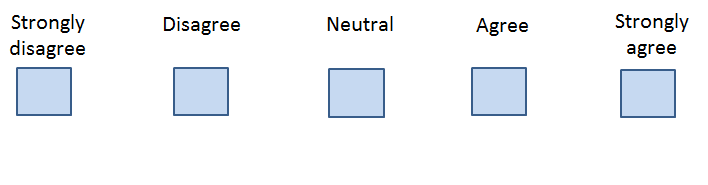

Supplement: S1 File — (DOCX) [file pone.0230832.s001.docx]
