# Supplementary material for: Education as a tool for improving canine welfare: Evaluating the effect of an education workshop on attitudes to responsible dog ownership and canine welfare in a sample of Key Stage 2 children in the United Kingdom
Source: PLoS One. 2020 Apr 20;15(4):e0230832. doi: 10.1371/journal.pone.0230832 (PMC7170237; doi:10.1371/journal.pone.0230832)
Supplement: S3 File — (DOCX) [file pone.0230832.s003.docx]

***This Lesson plan is intended for reference only. For any enquiries related to intended use please contact*** [***educ@dogstrust.org.uk***](mailto:educ@dogstrust.org.uk) ***for further information.***

| **Learning Outcomes** | **Assessment Methods** |
| --- | --- |
| Learners can **explain** what determines how friendly a dog is, and how safe a situation is. | Discussion using PP slides and breed cards |
| Learners can **identify** situations when it is not safe to stroke a dog, with reasoning | Worksheet and discussion of a range of scenarios. |
| Learners can **discuss** ways to adapt their behaviour to keep both themselves and their dog safe, happy and comfortable | Discussion using ‘Scenario Cards’ |
| **1 hour** |  |

| **Time** | **Delivery/Activity** | **Resources** | **Learning Outcomes** |
| --- | --- | --- | --- |
| 10 mins | **Educator :** Introduce self and   - Introduce the work of the organisation - Open a brief discussion about why you haven’t brought a real dog - Ask learners to raise their hands if they have a dog at home. Ask learners to raise their hands if they have every been told by their adults that they cannot have a dog, ask selected learners why and generate brief discussion around if dog ownership is suitable for everyone. - Open a brief discussion about the purpose of the workshop and why the Educator is there |  |  |
| 10 mins | **Educator:** Using images of different breeds of dogs, ask learners to vote on which of the breed they think is the friendliest (using “don’t know” or “neither” as an option as well). Avoid using words like “safest to stroke” because a) we are discouraging children from stroking any unknown dogs, and b) the word “safe” is slightly different to “friendly”. Their perception of what makes a dog “friendly” is a more important focus.  A dog could become unfriendly irrespective of its breed as a result of our behaviour, as well as the situation the dog is put in/if the owner is there/previous experiences/how it generally feels that day/if it is feeling poorly etc,. even if it normally doesn’t behave like that.  **Learners:** Vote on their choice of breed (or “don’t know”/”neither” option) and explain their initial reasoning.  **Key Questions during learner feedback:**   - *Is it true or fair to say that all children or people with brown hair (an element of their appearance) are unfriendly?* - *Are ALL Labradors (for example) safe?* - *Are ALL Staffies (for example) unfriendly?* - *Do all dogs have teeth? Could any dog therefore bite?* - *What IS biting? (a form of communication that is used by a dog when they feel they have no other choice)* - *What does affect whether a dog is friendly or not? (previous experiences, training, socialisation, if owner is present, individual personality, how they are feeling about the situation they are in (tied up for example, or lots of noise etc), and most importantly* ***our behaviour****).* - *Should we judge whether a dog is friendly based on its appearance or breed?* *So, what IS the most important thing to think about and focus on then?*   **Learners:** Through answering key questions, learners explain what determines how friendly a dog is, or how safe a situation is, using examples such as the dog’s previous experiences, if owner is present, individual personality, how they are feeling about the situation they are in (tied up for example, or lots of noise etc), and most importantly our behaviour).  **Educator:** Recap key points and generate brief summarising discussion | Images (laminated cards or slides) of different breeds/types of dogs including some culturally regarded as cute breeds and some regarded culturally as less cute | Learners can **explain** what determines how friendly a dog is, and how safe a situation is. |
| 10 mins | **Educator:** Introduce and explain the scenarios activity to the class telling them that for each scenario they need to think about 3 things (what they feel like doing, what someone else their age may feel like doing and what the safe thing to do is) and then record it on the recording sheet.  **Learners:** Participate in activity and record their answers on the recording sheet.  *For any delivery approach used (options discussed below), when explaining the activity,* ***it is essential to model the process****, explicitly giving the children ‘permission’ to feel like doing something unsafe. The children may appreciate a non dog-related example e.g., I might FEEL like eating a whole chocolate cake for lunch, but I don’t DO it because I know I will feel sick!*  *While it is important to encourage the children to be 100% honest, the ‘what a child my age would feel like doing’ option offers a distancing mechanism that allows the children to discuss the unsafe options without feeling uncomfortable admitting they would do that.*  ***Approaches to delivery -*** *There are various ways to deliver this activity but the time learners spend discussing scenarios by themselves should be minimal (apx 2 mins) - this needs to be an educator-led activity. It is preferable that groups are only given one scenario each and a few minutes to discuss. If they finish particularly quickly they can be given another scenario. It doesn’t matter if more than one group have the same scenario (if you have more than 4 groups, which is likely) as it will potentially identify different perceptions and interpretations.*  *Alternatively, it is perfectly fine to just show each scenario at a time, ask learners to discuss in pairs for 1 minutes, and then discuss in detail as a group, so the ‘Recording Sheet’ and group work approach doesn’t have to be used.*  **Educator**: Introduce the teasing scenario as the example for the activity.  Read, or ask a child to read the scenario from the slide and ask learners to consider the following Key Questions:  **Key Questions (teasing):**   - *What is teasing? How does it feel if someone teases us?* - *Can you think of any examples of what teasing a dog might look like? (try to encourage discussion around the obvious examples i.e. dressing them up, sitting on them etc, as well as the less obvious ones outlined in the* ***Conclusive Messaging*** *section below)* - *If we did tease our dog, what could happen? Who would have caused that to happen? How might it affect our relationship with the dog?* - *Is it ever OK to tease a dog? What would be better ways to interact with them? (training, praise, play…)* - *What should you do if you see someone else teasing a dog?* - *What should you do if somebody asks you to tease your dog, or asks if they can, because they think it’s harmless fun?*   **Conclusive Messaging (teasing):**  It’s crucial that learners have an in depth understanding of what teasing is in the first place in order for them to recognise when they themselves may be partaking in teasing, NB Dogs Trust regards teasing as anything that someone does around a dog that they may be enjoying or finding funny, but that the dog does not. They may have previously believed these behaviours to be benign or harmless fun, e.g. Putting antlers on them at Christmas because it’s “cute” or “funny”, picking up their front paws and dancing with them, continually commanding the same trick repeatedly without rewarding, general over handling/picking them up etc. Be clear that what may seem like harmless, innocent fun to us, is not seen that way by a dog and often they are worried by these unfair, confusing human behaviours. | Cards outlining 6 different scenarios a child may find themselves in where tempted to behave in the wrong way with a dog; being encouraged to tease a dog, a dog tied up without an owner, a dog eating, a dog sleeping or resting, being encouraged to hug a dog tight, a very young child being left alone with a dog.  Worksheet asking learners to record the three questions;   - what do I feel like doing, - what might someone else my age feel like doing - what is the safe thing to do | Learners can **identify** situations when it is not safe to stroke a dog, with reasoning. |
| 15 mins | **Educator:** Ask groups to present their responses to the scenario they were given/scenario discussed in pairs, inviting other class members to respond if they had any different ideas.  Explain messages relating to each scenario, and encourage children to always consider the following first of all for any situation involving dogs:   - *If you want to stroke a dog, first look at what it is doing. Are you going to be disturbing it?* - *How is the dog going to feel, and then potentially react, if you start stroking it? Who would have been responsible for causing that reaction* - *If it is not your dog, it is always best to leave them alone.*   **Key Questions (dog tied up without owner):**   - *How might a dog feel if it has been tied up and can’t see its owner?* - *How might it then feel if a stranger (so you or I) were to approach them?* - *If dogs feel worried, what behaviours do they display that tell us that?* - *If they weren’t tied up, what might their first choice be in terms of how they respond (would probably move or run away)?* - *Are they able to use that option in this situation?* - *How might they therefore be forced to communicate their worry? (essentially we are talking about the potential for biting – remember we explain biting as a* ***form of communication*** *that in this instance would have been caused by our behaviour)* - *As their choices are limited in terms of how they can respond to our behaviour, and their worry is likely to therefore build more quickly, why is it* ***both*** *unfair* ***and*** *unsafe to approach dogs that are tied up or restricted?*   **Conclusive Messaging (dog tied up):**  The main point here is that we should never approach dogs we don’t know as often they are just not interested in meeting strangers. In this particular situation the dog is tied up so the dog’s options in terms of how it responds to us are limited. The dog would be unlikely to consent to us touching them, and would be likely to feel worried and trapped if we were to approach. If we were to get too close, and the dog was then bite us through worry/fear, it would be our behaviour that caused that reaction.  **Key Questions (dog eating):**   - *Even if a dog doesn’t growl/bite, is it fair to touch them when they’re eating?* - *If you’re eating would you like it if someone bothered or disturbed you? What might you do if someone did do that, especially if you were eating your favourite food (i.e. shout at that person)? What might be the dog equivalent of that behaviour?*   **Conclusive Messaging (dog eating):**  Dogs must be left alone to eat in peace as that is the kindest, fairest way for us to behave. Dogs can sometimes feel protective over important resources like food, and may display that through growling, snarling and biting, if we get too close. That reaction would have been caused by our behaviour.  **Key Questions (dogs sleeping or resting):**   - *Have you ever had a bad day, go to your bedroom, shut your door and just want to be on your own? Is that the same as a dog going off to their bed?* - *If the dog is sleeping/resting somewhere else in the house, not in his bed, is it ok to touch him/her?* - *If you are sound asleep and somebody suddenly wakes you up and startles you, how might you feel and respond? What might the dog version of that reaction be?*   **Conclusive Messaging (dog in bed):**  Dogs must be left alone when sleeping or resting anywhere in the house, not just in their beds. They need to sleep for much longer than we do so it’s important they are left undisturbed. If a dog is resting or sleeping, that itself is an indication they do not wish to interact. There may be times a dog has gone to its bed/area, or even somewhere different to normal, because it is worried and trying to remove itself from a situation, or possibly because it doesn’t feel well.  **Key Questions (hugging):**   - *Why do people hug each other?* - *Do dogs know what hugs mean? Do they understand we’re trying to show we love them?* - *Also mention dogs’ eyesight close up isn’t great so person hugging would appear blurry- scary for dog* - *If we are a dog do they have a choice and have they given their consent?*   **Conclusive Messaging (hugging):**  The main point to conclude from hugging is that it is not a natural way for dogs to receive love/affection, and it removes their choice/consent. If a dog is worried by something we are doing, and hugging is likely to be one of those things for many dogs, they will try to move away. Hugging removes that option for them and could therefore cause their worry/anxiety/frustration to build, resulting in them reacting (i.e. growling/biting).  **Key Questions (children left alone with dogs):**   - *What sort of things might the toddler do to the dog?* - *Is it safe to ever leave a child alone with a dog? What if it’s their own dog?*   **Conclusive Messaging (children left alone with dogs):**  The conclusive point to draw out here is that children’s behaviour is not always sensible around dogs. This can be reached by exploring the concept of the toddler’s behaviour being potentially triggering of a dog bite and highlighting the fact that children (particularly aged three and below) are statistically far more likely to be bitten than adults because of this. The audience obviously are not in charge of supervision decisions but the point we are trying to enable them to understand is that dog bites to children are often caused by less sensible and less mature behaviour. Its less about parents always being around (though of course that is one of our key messages,) and more to do with gaining their understanding as to why adult supervision is necessary, even for themselves, and perhaps allowing them to reflect on their own less sensible behaviours.  **Learners:** Feed back and contribute to class discussion. |  | Learners can discuss ways to adapt their behaviour to keep both themselves and their dog safe, happy and comfortable |
| 5 – 10 mins | **Educator:** Introduce the situation of seeing a dog they don’t know that they would like to stroke. Explain that it is best not to stroke dogs that we see out and about at all.  Ask for several volunteers to role play how to stroke a dog safely. Guide learners through each step, whilst questioning why it is necessary. NB: it must be reiterated that **our** preference is that they don’t stroke unfamiliar dogs **at all.**  **Key Questions:**   - *Why is it generally fairer not to stroke dogs we don’t know? How might it make them feel and what might they be busy doing and enjoying that we would be interrupting?*   ***If we decide to still approach the dog and owner:***   - *Why can’t we just stroke the dog straight away?* - *Why should we let the dog approach us before touching them?* - *Why is it important the dog has the chance/choice to sniff us before we touch them?* - *If the dog stays where he/she is, or moves away, what does that mean and how should we then respond?* - *Why do we need to stay quiet and calm?*   **Learners:** Participate in discussion | Fake dog | Learners can **identify** situations when it is not safe to stroke a dog, with reasoning |
| 5 mins | **Educator:** Ask learners what they would do if a dog was not with its owner, and running towards them, including what to do if holding something of interest to the dog. Demonstrate the safest response would be to stand still, look away from the dog and throw away any item that the dog may be interested in such as a toy or food.  **Key Questions:**   - *Would it be OK to touch the dog? Why not?* - *What would happen if we ran away/screamed?* - *Why is it important to throw away any item we are holding that the dog shows interest in?*   **Learners:** Respond to educator’s questions, with explanations, and then discuss and demonstrate the safe and appropriate steps to follow.  **Educator:** Ask learners what they would do if a dog knocked them over.  **Key Questions:**   - *How might screaming make the dog feel, and then behave?* - *If we were to run away, what might the dog think is happening, and might it do in response?* - *If we are holding something of interest to a dog, such as food, why must we always just throw it as far away from us as we can?*   **Learners:** Participate in discussions and demonstrations. |  | Learners can **discuss** ways to adapt their behaviour to keep both themselves and their dog safe, happy and comfortable |
| 5 mins | **Educator:**  Conduct behaviour change related plenary assessment. Hand out post it notes to each learner. Ask them to write down at least one thing they will now do differently as a result of the workshop.  Invite selected learners to read out their statement and lead final discussion related to these. | Post it notes/ Whiteboards | Assessment of all LO |
