# Supplementary material for: Education as a tool for improving canine welfare: Evaluating the effect of an education workshop on attitudes to responsible dog ownership and canine welfare in a sample of Key Stage 2 children in the United Kingdom
Source: PLoS One. 2020 Apr 20;15(4):e0230832. doi: 10.1371/journal.pone.0230832 (PMC7170237; doi:10.1371/journal.pone.0230832)
Supplement: S4 File — (DOCX) [file pone.0230832.s004.docx]

***This Lesson plan is intended for reference only. For any enquiries related to intended use please contact*** [***educ@dogstrust.org.uk***](mailto:educ@dogstrust.org.uk) ***for further information.***

| **Learning Outcomes** | **Assessment Methods** |
| --- | --- |
| Learners will be able to **describe, with reasoning,** what owners must provide for a dog in order for them to be happy and healthy. | Mind map followed by verbal discussion |
| Learners can **explain** why different breeds or types of dogs require different types of enrichment and care. | Verbal discussion during bag of needs activity.  Dog/ breed laminated card sorting activity. |
| Learners can **explain and reflect** on whether dog ownership is suitable for everyone. | Verbal discussion.  Post it note plenary. |

| **Time** | **Delivery/Activity** | **Resources** | **Learning Outcomes** |
| --- | --- | --- | --- |
| 5 – 10 mins | **Educator :** Introduce self and   - Introduce the work of the organisation - Open a brief discussion about why you haven’t brought a real dog - Ask learners to raise their hands if they have a dog at home. Ask learners to raise their hands if they have every been told by their adults that they cannot have a dog, ask selected learners why and generate brief discussion around if dog ownership is suitable for everyone. - Open a brief discussion about the purpose of the workshop and why the Educator is there |  | Learners can **explain and reflect** on whether dog ownership is suitable for everyone. |
| 10 mins | **Educator :** Explain that if a dog is happy and healthy it is *likely* to be a better family pet and *can* make a great companion. This is heavily dependent, however, on the dog’s needs being well met, as well as our behaviour around them. We need to understand what a big commitment dog ownership is, and that having a dog is not suitable for everybody’s home and lifestyle.  Instruct learners to complete a mind map with a list of as many things they can think of that a dog needs to be safe, happy and healthy. Give them 1 minute, with a guideline of 8 ideas. This activity can be completed as a paired discussion, writing notes/ideas on whiteboards or on scrap paper.  **Learners:** Complete a mind map and share one of their responses with educator and class.  (Educator : Whilst children are completing their mind maps/discussions, place a breed card face down in front of each pair/table (depending on size/behaviour of the group). Try to select the cards with the breeds that children have said they own/know e.g. give the Collie owner the Collie breed card. Ask children not to turn the cards over until instructed).  **Key Questions during feedback:**  During the mind map feedback, the Educator should select some pairs of children to question with the following:  *“What might happen if a dog wasn’t given this – how might it feel, how might it begin to behave?”*  *“Whose fault would it be for the dog feeling and behaving this way?”*  *“What sometimes happens when dogs don’t behave in a way an owner would like?”* (This relates to relinquishment).  **Conclusive Messaging:**  Educator to ask class, *do all dogs need to be given these things?* YES (Challenge those who say no.)  **We have only scratched the surface, these are just some of the things dogs need to be happy and healthy, but in reality the list is extensive – showing us just how much of a commitment it can be to have a dog, that not everybody would be able to provide a dog with these things, and consequently, that there are lots of things to think about before getting one.**  Educator to ask class, do all dogs need the same amount of these things? E.g food/exercise? NO (Challenge those who say yes.)  **Even if our homes and lifestyles are suitable for a dog, we need to remember that different breeds/types may have differing needs, and not every breed/type will be suitable for our home and lifestyle. So as well as thinking about the commitment involved in being responsible for *any* dog, we also need to consider the specific breed or type we are getting and whether we can ensure *that* dog is happy and healthy.**  These are the ideas we are going to be exploring throughout the workshop. | Paper/Whiteboard  Pencils/pens.  Laminated Cards Showing Different Breeds | Learners will be able to **describe with reasoning** what owners must provide for a dog in order for them to be happy and healthy.  Learners can **explain and reflect** on whether dog ownership is suitable for everyone. |
| 25 mins | **Educator** : Ask learners to turn over the card in front of them. Explain that this is going to be the dog they are responsible for throughout the workshop and that they will be using it for the next activity. Using slide 6 introduce the activity,  **Activity:**  There are various way to deliver this main section.  For example, items in the bag of props can be presented through a game of charades, (but with the educator picking the items from the bag for the learner to act out). Alternatively, learners can take turns to sit at the front and be blindfolded to guess an item while three other class members give verbal clues to help guess the item. It is important, however, that whichever variation is chosen, it is the educator that decides the item so that messaging can be prioritised rather than left to chance.  After discussing each item, ask learners to hold up their card if they think their dog would need that item in their life.  **(NB: All learners should hold up their card, challenge those who don’t- e.g. why do you think a Chihuahua doesn’t need exercise?)**  Then, ask each pair to hold up their card if they think their dog would need more of that item/need than others, showing that different breeds may require different types of care.  These items/needs must be discussed first and prioritised – in whatever order:   - Exercise (Item could be lead/collar/harness) - Enrichment (Item could be kong/toy/snuffle mat) - Training (Item could be treats, clicker) - Diet (Item could be bowl/food)   Other items if time:   - Vet Care (Item could be stethoscope etc) - Grooming (Item could be brush)   For each item, key questions must be asked as outlined in the guidance document linked above, and any reference to safe behaviour that is relevant should be included.  Educator : Summarise activity with the below messaging included and use PP slide 7 to highlight *some* of the key points for children to remember.  **Conclusive Messaging:**  If we don’t give dogs the things they need, it is inevitably going to affect how they are feeling and will consequently affect their behaviour. This often ends up with dogs being relinquished to Dogs Trust when it is not their fault, or dogs just generally not being happy and healthy in their homes.  If your dog is showing, or starts to show, any of these behaviours, think about why. Think about and discuss what action you can take, and changes you can make to help your dog with the help of your grown ups.  Before getting a dog, think about whether your home and lifestyle is suitable for having a dog at all, and make sure you do plenty of research, from the right sources, to make sure that your home is right for the dog you would like. | Bag of responsible dog ownership props  Blindfold | Learners can **explain** why different breeds or types of dogs require different types of enrichment and care. |
| 5 mins | **Educator** : Conduct behaviour change related plenary assessment. Hand out post it notes to each learner. Ask them to write down at least one thing they will now do differently as a result of the workshop.  Invite selected learners to read out their statement and lead final discussion related to these. | Post its notes/Whiteboards | Assessment of all LO’s |
| 5mins | Q and A (if time allows) |  |  |
