# Supplementary material for: Education as a tool for improving canine welfare: Evaluating the effect of an education workshop on attitudes to responsible dog ownership and canine welfare in a sample of Key Stage 2 children in the United Kingdom
Source: PLoS One. 2020 Apr 20;15(4):e0230832. doi: 10.1371/journal.pone.0230832 (PMC7170237; doi:10.1371/journal.pone.0230832)
Supplement: S5 File — k, number of parameters; lokLik, log likelihood; AIC, Akaike’s information criterion; ΔAIC, difference in AIC compared with the model with the lowest AIC; wi, model weight; Retained, models within delta-6 AIC are not retained if they are more complex versions of nested models with better AIC support; Treatment, Random assignment of learner in to either treatment or control group; FSM, Free school meal percentage for participant’s class; Year, year group of participant; Gender, gender of participant; DO, dog ownership status of participant. (DOCX) [file pone.0230832.s005.docx]

| **Model** | **Description** | **k** | **logLik** | **AIC** | **ΔAIC** | **W_j_** | **Retained** |
| --- | --- | --- | --- | --- | --- | --- | --- |
| model10 | (Treatment) + (Gender) + (1\|Class) | 7 | -2771.96 | 5557.91 | 0 | 0.63 | ✔ |
| model6 | (Treatment) + (DO) + (Gender) + (1\|Class) | 10 | -2770.36 | 5560.72 | 2.81 | 0.16 | × |
| model9 | (Treatment) + (Year) + (1\|Class) | 8 | -2772.43 | 5560.86 | 2.95 | 0.14 | × |
| model4 | (Treatment) + (FSM) + (Year) + (1\|Class) | 11 | -2770.93 | 5563.85 | 5.93 | 0.032 | × |
| model12 | (Treatment) + (1\|Class) | 4 | -2778.72 | 5565.45 | 7.53 | 0.015 |  |
| model2 | (Treatment) + (FSM) + (Gender)*Year + (1\|Class) | 14 | -2769.36 | 5566.72 | 8.80 | 0.0077 |  |
| model3 | (Treatment) + (DO) + (Gender)*Year + (1\|Class) | 12 | -2771.42 | 5566.84 | 8.92 | 0.0073 |  |
| model11 | (Gender) * Year + (1\|Class) | 5 | -2779.48 | 5568.96 | 11.04 | 0.0025 |  |
| model1 | (Gender)*Year + (FSM) + (DO) + (Treatment) + (1\|Class) | 15 | -2769.90 | 5569.80 | 11.88 | 0.0017 |  |
| model7 | (Treatment) + (DO) + (Year) + (1\|Class) | 8 | -2776.96 | 5569.93 | 12.01 | 0.0015 |  |
| model8 | (Treatment) + (Gender)*Year + (1\|Class) | 6 | -2780.04 | 5572.08 | 14.17 | 0.00053 |  |
| model13 | (Year) + (1\|Class) | 6 | -2830.09 | 5672.19 | 114.27 | 9.71 |  |
| model17 | 1+ (1\|Class) | 3 | -2835.59 | 5677.19 | 119.27 | 7.97e-27 |  |
| model15 | (FSM) + (1\|Class) | 6 | -2833.08 | 5678.16 | 120.24 | 4.90e-27 |  |
| model5 | (Treatment) + (FSM) + (Gender) + (1\|Class) | 10 | -2829.36 | 5678.72 | 120.80 | 3.70e-27 |  |
| model16 | (DO) + (1\|Class) | 4 | -2836.19 | 5680.39 | 122.47 | 1.61e-27 |  |
| model14 | (Gender) + (1\|Class) | 4 | -2836.32 | 5680.63 | 122.71 | 1.42e-27 |  |
