# Supplementary material for: Education as a tool for improving canine welfare: Evaluating the effect of an education workshop on attitudes to responsible dog ownership and canine welfare in a sample of Key Stage 2 children in the United Kingdom
Source: PLoS One. 2020 Apr 20;15(4):e0230832. doi: 10.1371/journal.pone.0230832 (PMC7170237; doi:10.1371/journal.pone.0230832)
Supplement: S6 File — k, number of parameters; lokLik, log likelihood; AIC, Akaike’s information criterion; ΔAIC, difference in AIC compared with the model with the lowest AIC; wi, model weight; Retained, models within delta-6 AIC are not retained if they are more complex versions of nested models with better AIC support; Treatment, Random assignment of learner in to either treatment or control group; FSM, Free school meal percentage for participant’s class; Year, year group of participant; Gender, gender of participant; DO, dog ownership status of participant. (DOCX) [file pone.0230832.s006.docx]

| **Model** | **Description** | **k** | **logLik** | **AIC** | **ΔAIC** | **W_j_** | **Retained** |
| --- | --- | --- | --- | --- | --- | --- | --- |
| model2 | (Treatment) + (FSM) + (Gender)*Year + (1\|Class) | 14 | -3907.73 | 7843.47 | 0 | 0.78 | ✔ |
| model1 | (Gender)*Year + (FSM) + (DO) + (Treatment) + (1\|Class) | 15 | -3908.13 | 7846.25 | 2.78 | 0.19 | × |
| model8 | (Treatment) + (Gender)*Year + (1\|Class) | 11 | -3914.52 | 7851.05 | 7.58 | 0.02 |  |
| model4 | (Treatment) + (FSM) + (Year) + (1\|Class) | 10 | -3916.62 | 7853.24 | 9.77 | 0.006 |  |
| model3 | (Treatment) + (DO) + (Gender)*Year + (1\|Class) | 12 | -3914.94 | 7853.89 | 10.42 | 0.0043 |  |
| model9 | (Treatment) + (Year) + (1\|Class) | 7 | -3923.41 | 7860.83 | 17.36 | 0.00013 |  |
| model7 | (Treatment) + (DO) + (Year) + (1\|Class) | 8 | -3923.85 | 7863.71 | 20.24 | 3.14 |  |
| model5 | (Treatment) + (FSM) + (Gender) + (1\|Class) | 8 | -3929.84 | 7875.69 | 32.22 | 7.86 |  |
| model10 | (Treatment) + (Gender) + (1\|Class) | 5 | -3935.53 | 7881.06 | 37.59 | 5.36 |  |
| model6 | (Treatment) + (DO) + (Gender) + (1\|Class) | 6 | -3935.98 | 7883.96 | 40.49 | 1.25e-09 |  |
| model12 | (Treatment) + (1\|Class) | 4 | -3939.71 | 7887.43 | 43.96 | 2.22 |  |
| model11 | (Gender) * Year + (1\|Class) | 10 | -3965.84 | 7951.68 | 108.21 | 2.47 |  |
| model13 | (Year) + (1\|Class) | 6 | -3976.70 | 7965.40 | 121.93 | 2.59 |  |
| model14 | (Gender) + (1\|Class) | 4 | -3987.36 | 7982.71 | 139.24 | 4.52e-31 |  |
| model15 | (FSM) + (1\|Class) | 6 | -3987.53 | 7987.06 | 143.59 | 5.14e-32 |  |
| model17 | 1+ (1\|Class) | 3 | -3993.28 | 7992.55 | 149.08 | 3.30e-33 |  |
| model16 | (DO) + (1\|Class) | 4 | -3993.69 | 7995.37 | 151.90 | 8.05e-34 |  |
